# Effects of minocycline on dendrites, dendritic spines, and microglia in immature mouse brains after kainic acid‐induced status epilepticus

**DOI:** 10.1111/cns.14352

**Published:** 2023-07-12

**Authors:** Xiaoyue Yang, Tianyi Li, Jie Liu, Hong Sun, Li Cheng, Xiaojie Song, Ziyao Han, Hanyu Luo, Wei Han, Lingling Xie, Li Jiang

**Affiliations:** ^1^ Department of Neurology Children's Hospital of Chongqing Medical University Chongqing China; ^2^ National Clinical Research Center for Child Health and Disorders Chongqing China; ^3^ Ministry of Education Key Laboratory of Child Development and Disorders Chongqing China; ^4^ Chongqing Key Laboratory of Pediatrics Chongqing China

**Keywords:** dendrite, immature brain, microglia, minocycline, status epilepticus

## Abstract

**Purpose:**

This study aimed to investigate whether minocycline could influence alterations of microglial subtypes, the morphology of dendrites and dendritic spines, the microstructures of synapses and synaptic proteins, or even cognition outcomes in immature male mice following status epilepticus (SE) induced by kainic acid.

**Methods:**

Golgi staining was performed to visualize the dendrites and dendritic spines of neurons of the hippocampus. The microstructures of synapses and synaptic proteins were observed using transmission electron microscopy and western blotting analysis, respectively. Microglial reactivation and their markers were evaluated using flow cytometry. The Morris water maze (MWM) test was used to analyze spatial learning and memory ability.

**Results:**

Significant partial spines increase (predominate in thin spines) was observed in the dendrites of neurons after acute SE and partial loss (mainly in thin spines) was presented by days 14 and 28 post‐SE. The postsynaptic ultrastructure was impaired on the 7th and 14th days after SE. The proportion of M1 microglia increased significantly only after acute SE Similarly, the proportion of M2 microglia increased in the acute stage with high expression levels of all surface markers. In contrast, a decrease in M2 microglia and their markers was noted by day 14 post‐SE. Minocycline could reverse the changes in dendrites and synaptic proteins caused by SE, and increase the levels of synaptic proteins. Meanwhile, minocycline could inhibit the reactivation of M1 microglia and the expression of their markers, except for promoting CD200R. In addition, treatment with minocycline could regulate the expression of M2 microglia and their surface markers, as well as ameliorating the impaired spatial learning and memory on the 28th day after SE.

**Conclusions:**

Dendritic spines and microglia are dynamically changed after SE. Minocycline could ameliorate the impaired cognition in the kainic acid‐induced mouse model by decreasing the damage to dendrites and altering microglial reactivation.

## Graphical abstract

Dendritic spines, synapses, and microglia and their markers dynamically change during epileptogenesis in immature mice. Minocycline ameliorates cognitive deficits in immature epileptic mice, altering the microglial and dendritic levels.

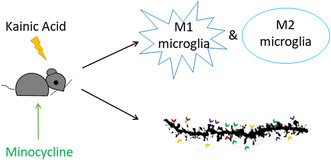

## 1 INTRODUCTION

Epilepsy, one of the most common and serious neurological diseases in the world, is clinically characterized by recurrent and spontaneous seizures, leading to neurobiological, cognitive, and psychosocial consequences.^1^ It is estimated that the prevalence of active epilepsy is 6.38 per 1000 individuals,^2^ and infants less than 1‐year‐old comprise the high‐incidence population for epilepsy.^3^ Furthermore, approximately one in four children with epilepsy experience cognitive comorbidity, and young age at onset (<5 years) is independently associated with cognitive defects.^4^ As the sites of the majority of excitatory synaptic inputs, neuronal dendrites and dendrite spines are strongly implicated in memory and cognition by affecting synaptic function and plasticity.^5^ Loss in dendritic spines and alterations in dendritic spine morphology have been reported in brain tissues both from patients with epilepsy and epileptic animal models.^6^, ^7^ However, despite considerable advances in epilepsy research, the pathogenetic mechanism between epilepsy and cognitive comorbidity remain unclear.

Microglia are the brain‐resident macrophages of innate immunity, which continuously surveil the environment of the central nervous system (CNS) to maintain homeostasis and the physiological function of brain.^8^ Traditionally, reactivated microglia are divided into M1 and M2 phenotypes: classic reactivated M1 microglia induce inflammation and neurotoxicity, while M2 microglia exert anti‐inflammatory and neuroprotective effects.^9^ Besides their innate immune activity, microglia also play a critical role in the sculpture of the neural circuits in the brain via phagocytosis of dendritic spines during the development period, according to recent evidence.^10^, ^11^, ^12^ Studies have demonstrated the reactivation of microglia in epilepsy, indicating the important role of microglia in epileptogenesis and cognitive outcomes.^13^, ^14^ In particular, the dynamic alterations of M1 and M2 microglia during different stages of epileptogenesis in the adult brain of epileptic animals have been reported, including our recent report.^13^, ^15^ Nonetheless, in the immature epileptic brain, the dynamic alteration of reactive microglia and the effects of the different microglial phenotypes still require further explanation.

As a highly brain‐penetrant tetracycline, minocycline has been shown to have anti‐inflammatory and neuroprotective properties in epileptic animal models.^16^, ^17^, ^18^ Several researches have reported the ameliorative effect of minocycline on cognition by influencing dendrite and dendritic spines^19^, ^20^ or microglia^21^ in different disease models. In the present study, we used a kainic acid‐induced status epilepticus (SE) mouse model to analyze the dynamic alterations of M1 and M2 microglia and the expression levels of several markers of microglial phenotypes and investigated the morphology of dendrites and dendritic spins in the hippocampus at different stages after SE in the immature brain. Moreover, we sought to determine whether minocycline treatment could affect microglia reactivation, dendrites and dendritic spines, or even cognition outcomes after SE. This data will be helpful to clarify the possible link between microglia in brain tissues and dendrites and dendritic spines in the hippocampus in the developmental brain in epilepsy.

## 2 MATERIALS AND METHODS

### 2.1 Animals

Postnatal 14‐day‐old (P14) immature male C57BL/6J mice were purchased from the Animal Resources Center of Chongqing Medical University (China). All the animals were housed and maintained on a 12/12‐h light/dark cycle with food and water ad libitum. To determine the dynamic changes of dendrites, dendritic spines, and microglia in immature mouse brains after kainic acid‐induced SE, we sacrificed the mice at three different time points, including 7, 14, and 28 days after SE and at the same points in the control (Ctl) group. To evaluate the effect of minocycline on SE, mice were assigned randomly into three groups: Ctl group, SE group, and SE with minocycline treatment (MINO) group. All animal experiments were approved by the Animal Ethics Committee of Chongqing Medical University and complied with their guidelines.

### 2.2 Status epilepticus model and drug treatment

The SE model was established in SE group and MINO group by intraperitoneal (i.p.) injection of kainic acid (1.5 mg/kg, Cayman Chemical Company). Diazepam (0.5 mg/kg, i.p., Kingyork Group) was administered 30 min after the beginning of seizures of score 3 to terminate the SE. After successful modeling, the mice in the MINO group were administered intraperitoneal injections of minocycline (10 mg/kg, Sigma–Aldrich) once a day for 3 consecutive days. The Ctl group mice received only 0.9% sodium chloride solution and diazepam. Mice were excluded from the study due to failure to induce SE or death after SE. The ratio of failure to induce SE and death after SE were 21% and 14%, respectively.

### 2.3 Isolation of brain microglia and flow cytometry

Microglia were isolated according to a previously described Percoll gradient centrifugation method,^22^ with slight modifications. Microglial cell surface markers were identified by flow cytometry (FC) according to a previous report.^23^ Isolated cells were initially blocked with rat serum for 30 min at 4°C and then stained for 20 min at 4°C in the dark with the following monoclonal antibodies: anti‐mouse CD45 molecule (CD45)‐Allophycocyanin (APC)/Cyanine 7 (Cy7), integrin subunit alpha M (CD11b)‐APC, Fc gamma receptor III (CD16/32)‐Phycoerythrin (PE), mannose receptor C‐type 1 (CD206)‐Peridinin chlorophyll protein complex (PerCP)/Cyanine 5 (Cy5), C‐X3‐C motif chemokine receptor 1 (CX3CR1)‐PE/Cy7, C‐X‐C motif chemokine receptor 3 (CXCR3)‐Brilliant Violet 510, CD68 molecule (CD68)‐Brilliant Violet 605, and CD200 receptor (CD200R)‐fluorescein isothiocyanate (FITC) (Biolegend; Table S1). The cells were then washed three times with 1 mL of 1 × PBS and resuspended in 200 μL of PBS for detection. Microglia were defined as CD11b^+^/CD45^int^, and M1 and M2 microglia were defined as CD11b^+^CD45^int^CD16/32^+^CD206^−^ and CD11b^+^CD45^int^CD206^+^ populations, respectively.^23^, ^24^ Cells were analyzed using a BD FACS Canto II cytometer (BD Bioscience), and 20,000 events were recorded. The cells were gated based on the appropriate negative isotype controls, and the data were analyzed using FlowJo Software X (TreeStar, Inc.).

### 2.4 Western blotting analysis

Total protein samples were extracted using a total protein extraction kit (BestBio) and the protein concentration was assessed using a Bio‐Rad protein assay kit (Bio‐Rad Laboratories). Samples containing 15 μg of protein were separated by 10% SDS polyacrylamide gel electrophoresis and transferred to a polyvinylidene difluoride membrane (0.22 μm, Millipore). The membrane was blocked for 1 h at room temperature in PBS containing 5% bovine serum albumin and further incubated with specific primary detection antibodies (anti‐synaptophysin (SYP), rabbit monoclonal antibody (Abcam; 1:50,000); anti‐growth‐associated protein 43 (GAP‐43), rabbit monoclonal antibody (Abcam; 1:100,000); anti‐postsynaptic density protein 95 (PSD‐95), goat polyclonal antibody (Abcam; 1 μg/mL); and an anti‐β‐actin antibody (1:500, Chemicon)) overnight at 4°C. Subsequently, peroxidase–conjugated secondary antibodies were applied to the same membranes for another 1 h at room temperature. Protein bands were visualized using clarity western electrochemiluminescence (ECL) substrate (Bio‐Rad) and analyzed by densitometry using ACDSee Pro2.5 (ACDSee, Inc.) and Image J software (NIH).

### 2.5 Golgi staining and analysis of dendrites

To visualize changes to neurons and dendritic spines, Golgi‐Cox staining was performed using an FD Rapid GolgiStain Kit (PK401, FD NeuroTechnologies) following the manufacturer's instructions. The whole brain was impregnated for 2 weeks at room temperature in the dark and transferred to solution C for 72 h subsequently. Then, sections of the brain (120 μm thick) were prepared in the coronal orientation.

The dendritic length, dendritic complexity, and spine morphology in the brain sections were analyzed as previously described^25^, ^26^ with slight modifications in our laboratory (Figure S1). Before the quantitative analysis, brain sections containing the dorsal hippocampus were coded until the analysis was completed. A 100× oil immersion objective (Nikon 100xA/1.25 Oil, Wuxi, China) was used to image the dendritic segments under brightfield illumination. The length of the dendrites on a 120‐μm‐thick section was calculated for each dendritic tree, and linear spine density was counted as the number of spines per 30 μm of dendrite length using Image J software as described previously.^27^ Sholl analysis was conducted to determine the dendritic complexity as number of branch points.^28^, ^29^ A final average was calculated for each of the three groups. Spine counts were averaged as the number of spine subtypes and total spines per 30 μm of dendritic length.

### 2.6 Transmission electron microscopy (TEM)

Mice were anesthetized using pentobarbital sodium (7.5 mg/mL, 0.05 mL/10 g i.p.), and then perfused with 0.9% sodium chloride and a mixture containing 4% paraformaldehyde and 0.5% glutaraldehyde. Hippocampal tissue blocks (1 mm^3^) were dissected and fixed in 4% dedicated glutaraldehyde for 2 h at 4°C, and then osmicated in 0.1 M phosphate‐buffered osmium tetroxide (OsO4) at 4°C for 2 h. After dehydration in an ascending ethanol and acetone series, the blocks were infiltrated and embedded with epoxy resin. A single 60 nm‐section was cut from each epoxy resin block and stained with uranyl acetate and lead citrate. To analyze the morphological changes of the hippocampal synapse at the ultrastructural level, a transmission electron microscope (JEM‐1400plus, JEOL, Ltd.) was used to examine the sections. For each section, three fields of vision were randomly chosen and photographed at magnifications of 30,000×. The length of the active zone (AZ) and the thicknesses of the postsynaptic density (PSD) were measured three times in the field of view (30,000× magnification) for each group using Image J software, the mean values of which were used to perform the statistical analysis.

### 2.7 Morris water maze tests

After 28 days of SE modeling, the Morris water maze (MWM) was used to test the spatial learning and memory abilities of the three groups of mice. During the acquisition trials, all mice were tested in four trials per day for five consecutive days. For each trial, the mouse was randomly placed into one of the four quadrants (N, S, E, and W). Each trial automatically ended when the mouse landed on a submerged goal platform for 3 s or after 60 s. If the mouse failed to find the platform within 60 s, it was gently guided there and allowed to remain in place for 20 s. The mean escape latency (the time taken to find the platform) of each mouse was recorded. On the last day of the spatial probe trial, the goal platform was removed, and then each mouse was placed in the maze from the farthest points from the location of the removed platform for 60 s. The mean time spent in the target region, and the mean number of times crossing the platform were measured for each mouse.

### 2.8 Statistical analysis

GraphPad Prism 8.0 software (GraphPad) was used for all statistical analyses.

The data were subjected to test for normality by Shapiro–Wilk test or Kolmogorov–Smirnov test. For data with a normal distribution, data were presented as the mean **±** standard error of mean (SEM), and the one‐way ANOVA or two‐way ANOVA with Dunnett's post hoc tests was employed. For data with an abnormal distribution, data were presented as the median (interquartile range), and the Kruskal–Wallis test was employed. *p* < 0.05 was considered the significance threshold for significance.

## 3 RESULTS

### 3.1 Dynamic changes in the levels of SYP, GAP‐43, and PSD‐5 proteins in the hippocampus after SE

**FIGURE 1:**
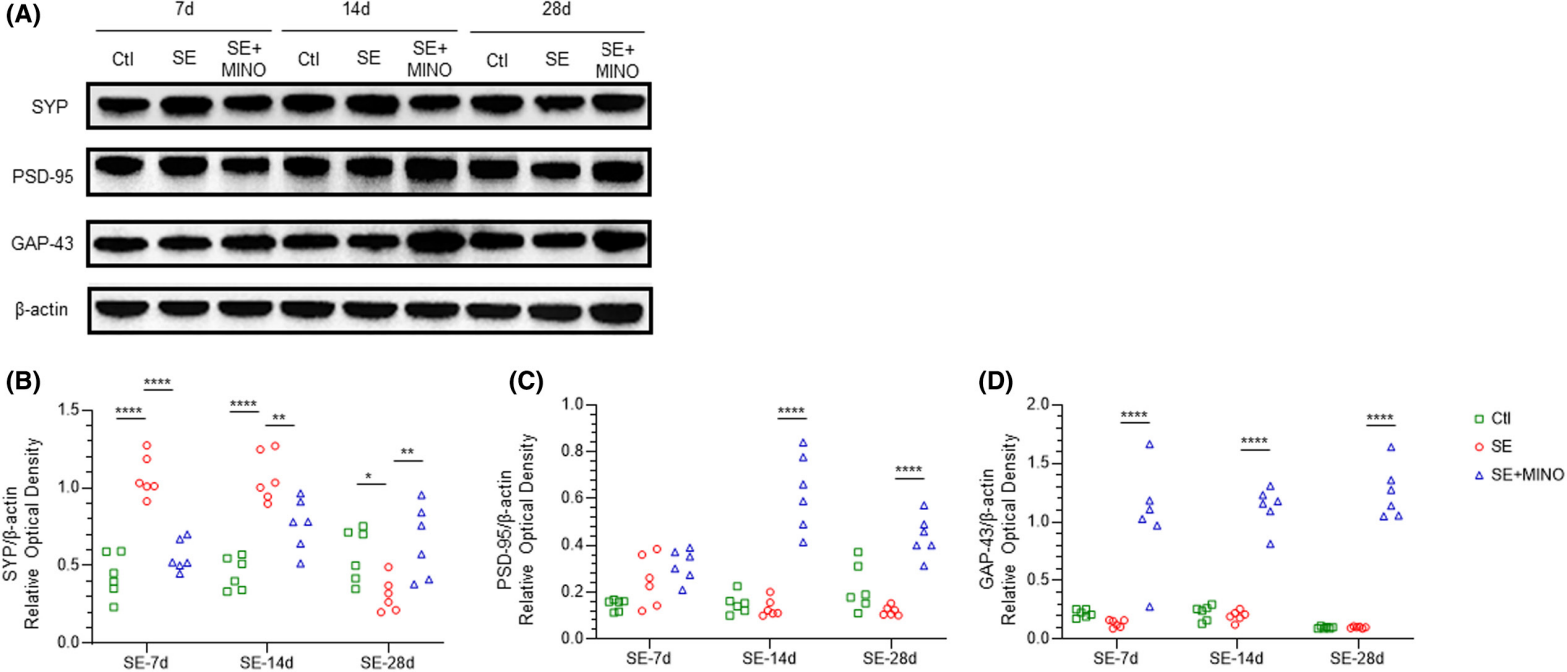
Effect of minocycline on protein expression levels of SYP, GAP‐43, and PSD‐95 in the hippocampus. (A) Western blotting analysis showing SYP, GAP‐43, and PSD‐95 protein levels in hippocampal tissue samples from mice on the 7th, 14th, and 28th days after KA‐induced SE. (B–D) Quantitative analyses of the SYP, GAP‐43, and PSD‐95 protein levels. One‐way ANOVA and Kruskal–Wallis test followed by Dunnett's post hoc test were used, *p < 0.05, **p < 0.01, ***p < 0.001, ****p < 0.0001. GAP‐43, growth‐associated protein 4; KA, kainic acid; PSD‐95, postsynaptic density protein 9; SE, status epilepticus; SYP, synaptophysin.

By western blotting, we analyzed the levels of SYP, GAP‐43, and PSD‐95 in the hippocampus to evaluate synaptic damage (Figures 1 and S4). The levels of the SYP increased significantly on the 7th (*F* = 42.70, *p*_Ctl vs SE_ < 0.0001) and 14th days (*F* = 26.73, *p*_Ctl vs SE_ < 0.0001) after SE compared to those in the Ctl; however, it decreased compared to the Ctl group (*F* = 5.957, *p*_Ctl vs SE_ = 0.0428) on the 28th day. However, on the 7th, 14th, and 28th days, no significant differences were found in the levels of GAP‐43 and PSD‐95 between the SE and Ctl groups. These results showed that the levels of SYP were increased in the acute and transitional stages, but decreased on the 28th day after SE. GAP‐43 and PSD‐95 levels did not differ after SE.

On the 7th and 14th day after SE, compared with that in the SE groups, the levels of SYP decreased markedly in the MINO group (day7: *F* = 42.70, *p*_SE vs SE + MINO_ < 0.0001; day14: *F* = 26.73, *p*_SE vs SE + MINO_ = 0.0053); moreover, it increased in the MINO group, to a level similar to that in the Ctl group on the 28th day (*F* = 5.957, *p*_SE vs SE + MINO_ = 0.0090). The levels of GAP‐43 in the MINO group were higher than those in the SE group at all time points (day7: *F* = 22.28, *p*_SE vs SE + MINO_ < 0.0001; day14: *F* = 142.1 *p*_SE vs SE + MINO_ < 0.0001; day28: *F* = 155.5, *p*_SE vs SE + MINO_ < 0.0001). The levels of PSD‐95 increased significantly in the MINO group in comparison with those in the SE group on the 14th and 28th days (day14: *F* = 47.02, *p*_SE vs SE + MINO_ < 0.0001; day28: *F* = 26.24, *p*_SE vs SE + MINO_ < 0.0001). Thus, minocycline could rescue the damage caused by SE on the expression of SYP by days 7, 14, and 28 post‐SE, and improve the GAP‐43 and PSD‐95 proteins levels after SE.

### 3.2 Changes of microglial phenotypes and expression of CX3CR1, CXCR3, CD68, and CD200R following SE

**FIGURE 2:**
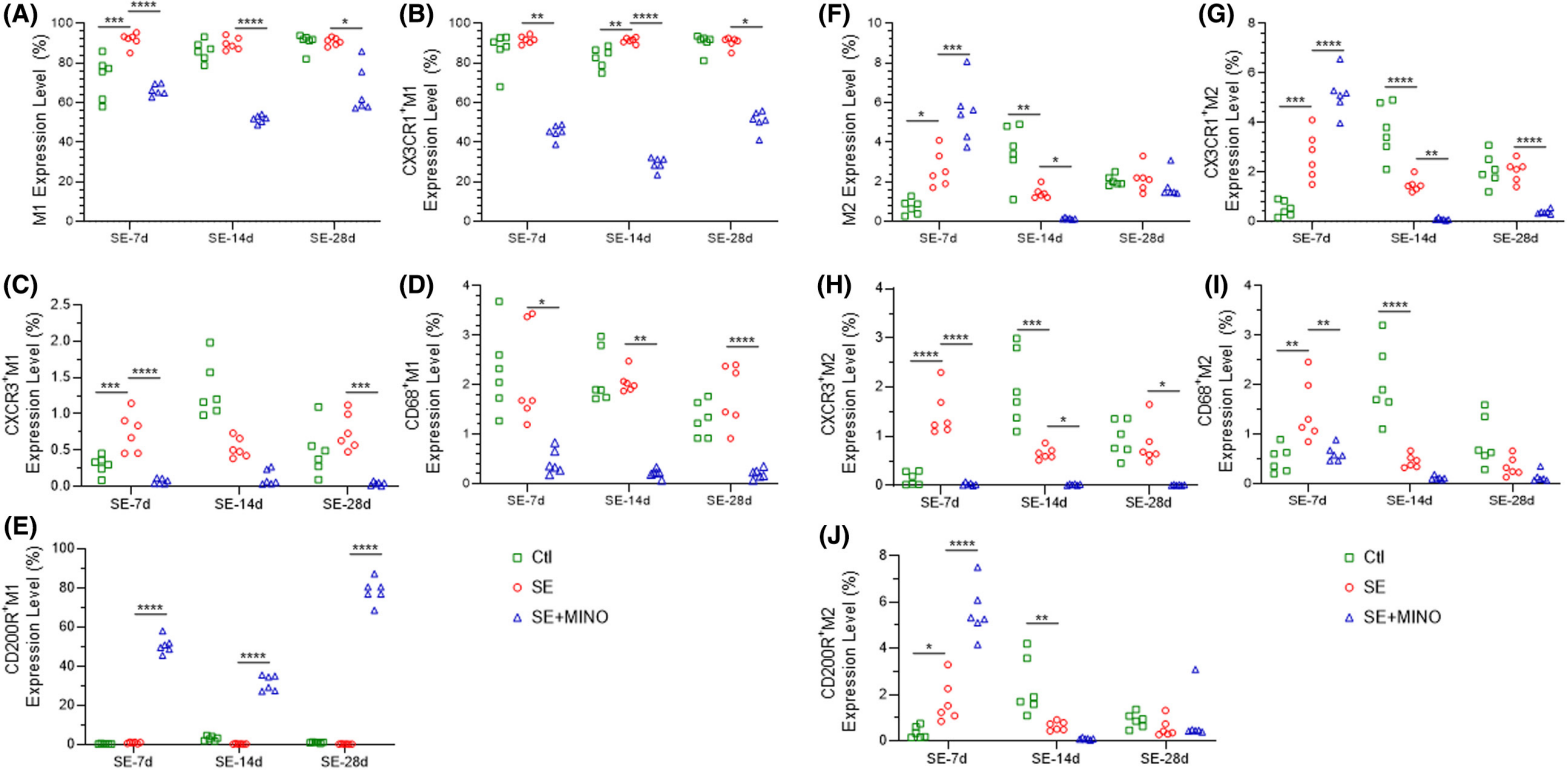
Expression levels of M1 and M2 microglia and microglial surface markers on the 7th, 14th, and 28th days after KA‐induced SE. (A) Expression levels in M1 microglia. (B–E) Proportion of CX3CR1, CXCR3, CD68, and CD200R levels on M1 microglia expression levels. (F) Expression levels in M2 microglia. (G–J) Proportion of CX3CR1, CXCR3, CD68, and CD200R levels on M2 microglia expression levels. One‐way ANOVA and Kruskal–Wallis test followed by Dunnett's post hoc test were applied, *p < 0.05, **p < 0.01, ***p < 0.001, ****p < 0.0001. CX3CR1, C‐X3‐C motif chemokine receptor 1; CXCR3, C‐X‐C motif chemokine receptor; CD68, CD68 molecule; CD200R, CD200 receptor 1; KA, kainic acid; SE, status epilepticus.

Changes in microglia phenotypes, the levels of CX3CR1, CXCR3, CD68, and CD200R, and levels of markers of the M1/M2 profile of microglia were investigated using flow cytometry. The expression of M1/M2 markers was quantified at the three time points (Figures 2 and S2). M1 microglia showed a significant increase by day 7 post‐SE (*F* = 22.97, *p*_Ctl vs SE_ = 0.0004) and this increase disappeared by day 14; moreover, no difference in the M1 phenotype was noted by day 28 post‐SE.

The protein markers of microglia were also changed after SE (Figure 2). Compared with the Ctl group, a dramatic increase in CXCR3 expression on M1 cells was observed by day 7 post‐SE (*F* = 23.54, *p*_Ctl vs SE_ = 0.0008); however, there were no significant differences between the groups for the expression of CX3CR1, CD68, and CD200R on M1 cells. By day 14 post‐SE, the level of CX3CR1 was significantly increased (*F* = 527.2, *p*_Ctl vs SE_ = 0.0016). Whereas there was no difference in the level of CXCR3 or CD68 or CD200R. By day 28 post‐SE, there were no significant differences in the levels of CX3CR1, CXCR3, CD68, and CD200R between the SE and Ctl groups.

The percentage of M2 cells was significantly increased by day 7 post‐SE (*F* = 32.19, *p*_Ctl vs SE_ = 0.0113), and then markedly decreased by day 14 post‐SE (*F* = 25.80, *p*_Ctl vs SE_ = 0.0010). Finally, the proportion of M2 cells increased to that in the Ctl group by day 28. Similarly, CX3CR1 (*F* = 56.73, *p*_Ctl vs SE_ = 0.0004), CXCR3 (*F* = 48.24, *p*_Ctl vs SE_ < 0.0001), CD68 (*F* = 10.66, *p*_Ctl vs SE_ = 0.0014), and CD200R (*F* = 60.46, *p*_Ctl vs SE_ = 0.0285) levels in M2 cells increased by day 7 post‐SE, and then significantly decreased by day 14 (*F*_CX3CR1_ = 47.92, *p*_Ctl vs SE_ < 0.0001; *F*_CXCR3_ = 29.93, *p*_Ctl vs SE_ = 0.0003; *F*_CD68_ = 32.26, *p*_Ctl vs SE_ < 0.0001; *F*_CD200R_ = 15.69, *p*_Ctl vs SE_ = 0.0019), finally increasing to the levels in the Ctl by day 28 post‐SE. These results indicated that, in the SE group, numerous microglia became reactive and mainly differentiated into the M1 phenotype, and both M1 and M2 microglia increased significantly during the acute period. Gradually, M2 microglia markedly decreased during the transitional period. Finally, M1 and M2 microglia were similar between SE and Ctl groups on the 28th day after SE. Accompanied by the changes in microglial phenotypes, there was a fluctuation in the expression levels of different microglial markers.

Based on these results, under the treatment of minocycline, by day 7 post‐SE, the percentage of M1 cells decreased significantly (*F* = 22.97, *p*_SE vs SE + MINO_ < 0.0001), and continued to decrease by days 14 (*F* = 203.1, *p*_SE vs SE + MINO_ < 0.0001) and 28 (*p*_SE vs SE + MINO_ = 0.0161) post‐SE compared with that in the SE group. Accompanied by M1 cells' alteration, the markers also decreased in the MINO group by days 7, 14, and 28 post‐SE (day7: for CX3CR1, *p*_SE vs SE + MINO_ = 0.0016; for CXCR3, *F* = 23.54, *p*_SE vs SE + MINO_ < 0.0001; for CD68, *p*_SE vs SE + MINO_ = 0.0161; day14: for CX3CR1, *F* = 527.2, *p*_SE vs SE + MINO_ < 0.0001; for CD68, *p*_SE vs SE + MINO_ = 0.0034; day28: for CX3CR1, *p*_SE vs SE + MINO_ = 0.0116; for CXCR3, *F* = 12.88, *p*_SE vs SE + MINO_ = 0.0003; for CD68, *F* = 22.65, *p*_SE vs SE + MINO_ < 0.0001), except for CD200R, which was increased at all the three time‐points (day7, *F* = 861.9 *p*_SE vs SE + MINO_ < 0.0001; day14, *F* = 319.8 *p*_SE vs SE + MINO_ < 0.0001; day28, *F* = 963.6, *p*_SE vs SE + MINO_ < 0.0001).

The proportion of M2 cells increased markedly with minocycline treatment compared with those in the SE group by day 7 (*F* = 32.19, *p*_SE vs SE + MINO_ = 0.0005), but was less than those in the SE group by day 14 (*F* = 25.80, *p*_SE vs SE + MINO_ = 0.0286). Moreover, there was no difference in M2 cells between the two groups by day 28 post‐SE. By day 7, significant increases in the CX3CR1 (*F* = 56.73 *p*_SE vs SE + MINO_ < 0.0001) and CD200R (*F* = 60.46 *p*_SE vs SE + MINO_ < 0.0001) expression on M2 cells of the MINO group were observed, while the CXCR3 (*F* = 48.24, *p*_SE vs SE + MINO_ < 0.0001) and CD68 (*F* = 10.66, *P*_SE vs SE + MINO_ = 0.0039) expression levels were decreased. By day 14, the CX3CR1 (*F* = 47.92, *p*_SE vs SE + MINO_ = 0.0035) and CXCR3 (*F* = 29.93, *p*_SE vs SE + MINO_ = 0.0476) levels were decreased comparing with those in the SE group, but there was no difference in the CD68 and CD200R levels between the two groups. Besides, by day 28, CX3CR1 (*F* = 27.92, *p*_SE vs SE + MINO_ < 0.0001) and CXCR3 (*p*_SE vs SE + MINO_ = 0.0161) levels were reduced in the MINO group, while there was no difference in CD68 and CD200R levels between the MINO and SE groups. Minocycline inhibited microglia reactivation and switched the M1/M2 phenotypes, as well as altered the expression levels of microglial markers after SE.

### 3.3 Changes in the morphology of dendrites and spines of granule and pyramidal neurons in the hippocampus

**FIGURE 3:**
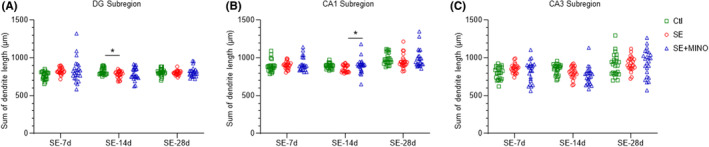
Total spine length of granule cells in the DG subregion (A) and pyramidal cells in the CA1 and CA3 subregions (B and C) of the hippocampus on the 7th, 14th, and 28th days after KA‐induced SE. One‐way ANOVA and Kruskal–Wallis test followed by Dunnett's post hoc test were applied, *p < 0.05. CA1, cornu ammonis 1; CA3, cornu ammonis 3; DG, dentate gyrus; KA, kainic acid; SE, status epilepticus.

**FIGURE 4:**
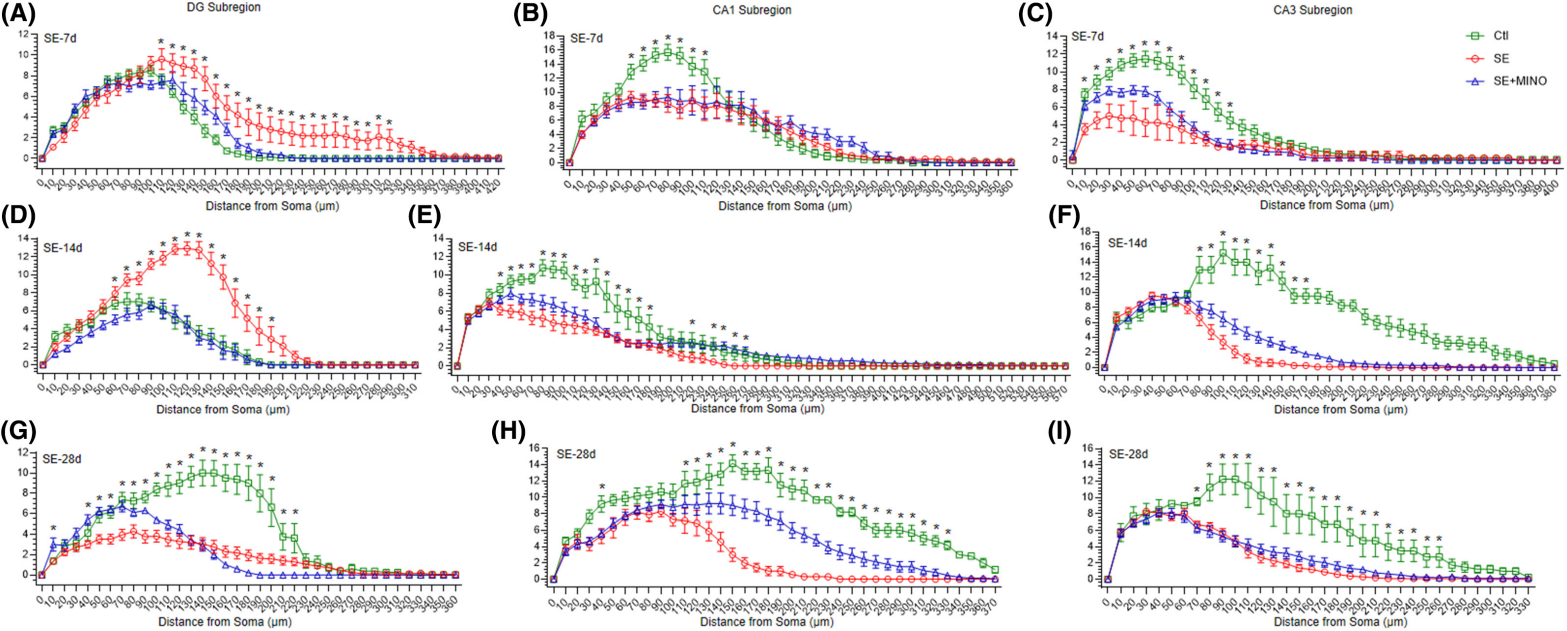
Dendritic complexity of granule cells in the DG subregion (A, D and G) and pyramidal cells in the CA1 (B, E and H) and CA3 subregions (C, F and I) of the hippocampus on the 7th, 14th, and 28th days after KA‐induced SE. Two‐way ANOVA with Dunnett's post hoc test was used, *p < 0.05. CA1, cornu ammonis 1; CA3, cornu ammonis 3; DG, dentate gyrus; KA, kainic acid; SE, status epilepticus.

**FIGURE 5:**
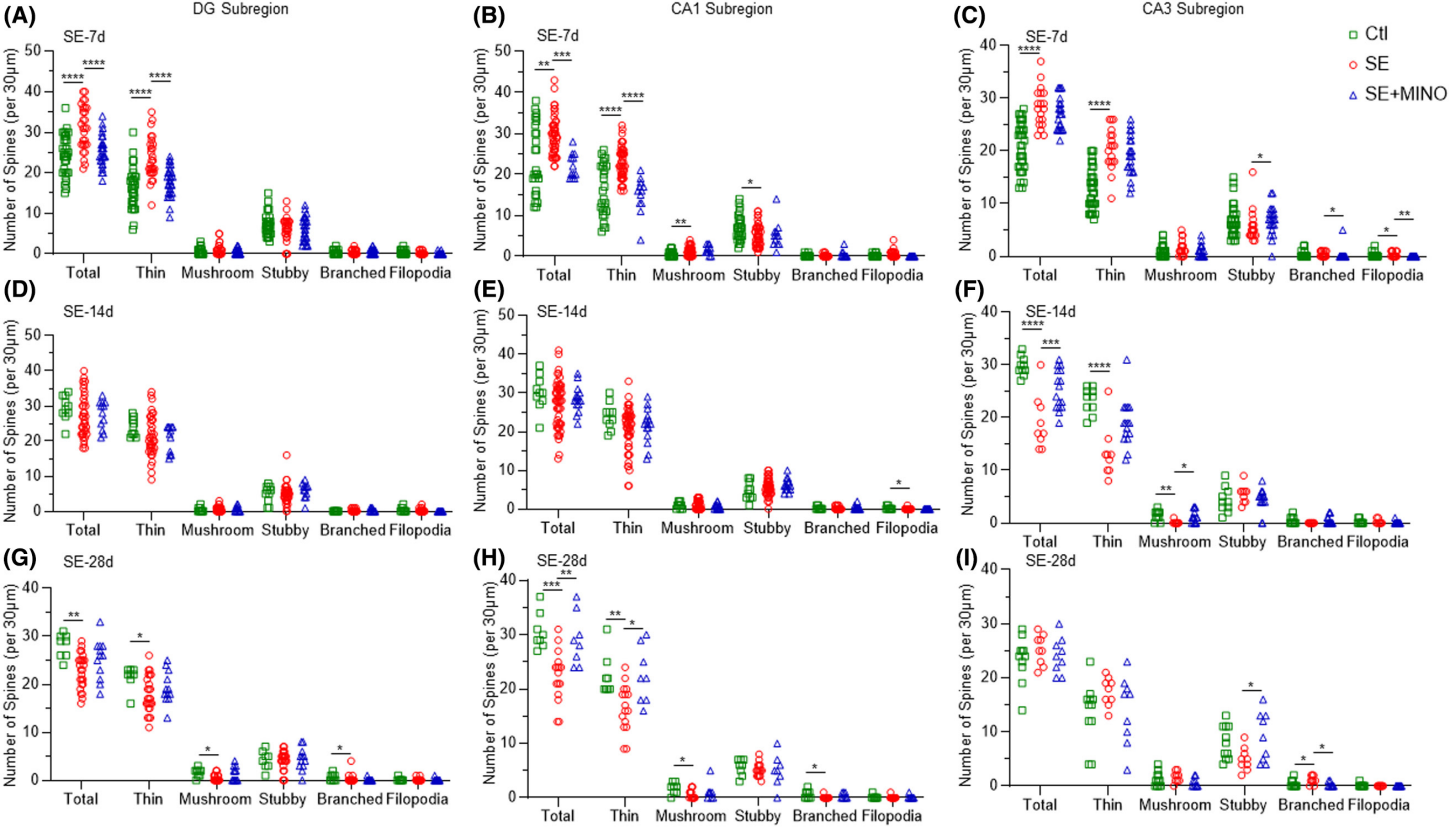
Number of different categories of spines per 30 μm of each dendrite in the DG, CA1, and CA3 subregions of the hippocampus on the 7th, 14th, and 28th days after KA‐induced SE. One‐way ANOVA and Kruskal–Wallis test followed by Dunnett's post hoc test were applied, *p < 0.05, **p < 0.01, ***p < 0.001, ****p < 0.0001. CA1, cornu ammonis 1; CA3, cornu ammonis 3; DG, dentate gyrus; KA, kainic acid; SE, status epilepticus.

To study the morphology of the dendrites and the spines of granule neurons of DG subregion, as well as pyramidal neurons of CA1 and CA3 subregions in the hippocampus, we performed Golgi‐Cox staining (Figure S3). Consequently, the dendrite length (Figure 3) and the complexity of the dendritic arbor (Figure 4) were analyzed, in addition to the dendritic spine morphology (Figure 5) in the above‐mentioned regions.

In the DG subregion, the total length of dendrites in SE group was significantly shorter than that in the Ctl group by day 14 post‐SE (*p*_Ctl vs SE_ = 0.0482), and no difference was observed by days 7 and 28 post‐SE. However, the SE group showed no difference in the total dendritic length in the CA1 and CA3 subregions comparing with those in the Ctl group at any observed time point. Minocycline only boosted the total length of dendrites in CA1 subregion by day 14 post‐SE (*p*_SE vs SE + MINO_ = 0.0316).

The complexity of the dendritic arbor was examined by performing Sholl analysis to determine the number of intersections within concentric circles every 10 μm from the soma to the distal ends of the dendrites. In the DG subregion, there were significant increases in the number of intersections of dendrites both on days 7 (*F* = 84.66, for 110–320 μm all *p*_Ctl vs SE_ < 0.05) and 14 (*F* = 127.6, for 80–190 μm all *P*_Ctl vs SE_ < 0.05) post‐SE, but on day 28 post‐SE it was markedly decreased comparing with that in the Ctl group (*F* = 214.2, for 50–220 μm all *p*_Ctl vs SE_ < 0.05). At all three stages after SE, the numbers of intersections of dendrites in the CA1 and CA3 subregions decreased significantly (CA1: *F*_day7_ = 10.7, for 50–110 μm all *p*_Ctl vs SE_ < 0.05; *F*_day14_ = 76.36, for 40–80 μm all *p*_Ctl vs SE_ < 0.05; *F*_day28_ = 254.0, for 40 μm and 110 μm to 330 μm all *P*_Ctl vs SE_ < 0.05. CA3: *F*_day7_ = 82.54, for 10–130 μm all *p*_Ctl vs SE_ < 0.05; *F*_day14_ = 442.4, for 80–320 μm all *p*_Ctl vs SE_ < 0.05; *F*_day28_ = 187.7, for 70–260 μm all *p*_Ctl vs SE_ < 0.05). In the MINO group, the number of intersections of dendrites in the DG subregion was decreased on the 7th (*F* = 84.66, for 100–110 μm, 130–290 μm, and 310–320 μm, all *p*_SE vs SE + MINO_ < 0.05), 14th (*F* = 127.6, for 60–190 μm all *p*_SE vs SE + MINO_ < 0.05), and 28th (*F* = 214.2, for 180–200 μm all *p*_SE vs SE + MINO_ < 0.05) days after SE; however, partly increased by day 28 (*F* = 214.2, for 10 μm and 40–90 μm all *p*_SE vs SE + MINO_ <0.05) post‐SE comparing those in the SE group. Furthermore, increases were observed in the number of intersections of dendrites in the CA1 subregion by days 14 (*F* = 76.36, for 50, 70–100, 220, and 240–270 μm, all *p*_SE vs SE + MINO_ < 0.05) and 28 (*F* = 254.0, for 130–250 μm all *p*_SE vs SE + MINO_ < 0.05) post‐SE, and the CA3 subregion by days 7 (*F* = 82.54, for 50–60 μm all *p*_SE vs SE + MINO_ < 0.05) and 14 (*F* = 442.4, for 90–160 μm all *p*_SE vs SE + MINO_ < 0.05) post‐SE.

The dendritic spine morphology was evaluated by counting the spine numbers of each selected dendrite according to the spine categories. In the DG subregion, the number of total spines was markedly increased by day 7 post‐SE (*F* = 16.20, *p*_Ctl vs SE_ < 0.0001), followed by a significant decrease by day 28 post‐SE (*F* = 7.299, *p*_Ctl vs SE_ = 0.0013). Similarly, among these spines, the number of thin spines was significantly increased by day 7 post‐SE (*F* = 19.44, *p*_Ctl vs SE_ < 0.0001), and then gradually decreased by day 14, followed by a dramatic decrease by day 28 (*p*_Ctl vs SE_ = 0.0255). Decreases in mushroom spines (*p*_Ctl vs SE_ = 0.0134) and branched spines (*p*_Ctl vs SE_ = 0.0350) by day 28 post‐SE were presented. However, there were no significant differences in the numbers of stubby or filopodia spines between the SE and Ctl groups at any time point.

In the CA1 subregion, the total number of spines increased significantly (*p*_Ctl vs SE_ = 0.0036) by day 7, but decreased (*F* = 11.43, *p*_Ctl vs SE_ = 0.0004) by day 28 post‐SE comparing with that of the Ctl group. Among these spines, the numbers of thin and mushroom spines were significantly increased by day 7 post‐SE (thin: *F* = 22.41, *p*_Ctl vs SE_ < 0.0001; mushroom: *p*_Ctl vs SE_ = 0.0071), followed by a dramatic decrease by day 28 (thin: *p*_Ctl vs SE_ = 0.0072; mushroom: *p*_Ctl vs SE_ = 0.0264). In contrast, a significant decrease in stubby spines in the CA1 subregion (*F* = 3.831, *p*_Ctl vs SE_ = 0.0181) was observed by day 7 post‐SE. Furthermore, decreases in filopodia spines by day 14 (*p*_Ctl vs SE_ = 0.0215) and branched spines by day 28 post‐SE (*p*_Ctl vs SE_ = 0.0348) were noted.

In the CA3 subregion, a dramatic increase in the total number of dendrites was observed by day 7 post‐SE (*F* = 28.42, *p*_Ctl vs SE_ < 0.0001), which decreased significantly by day 14 (*F* = 19.09, *p*_Ctl vs SE_ < 0.0001). Among these spines, the number of thin spines was significantly increased by day 7 post‐SE (*F* = 29.36, *p*_Ctl vs SE_ < 0.0001), and then gradually decreased by day 14 (*p*_Ctl vs SE_ < 0.0001). Significant differences between SE and Ctl groups were observed in the numbers of filopodia spines by day 7 (*p*_Ctl vs SE_ = 0.0263), mushroom spines by day 14 (*p*_Ctl vs SE_ = 0.0077), and branched spines by day 28 (*p*_Ctl vs SE_ = 0.0424) post‐SE.

With minocycline treatment, the total number of spines was decreased comparing with that in the SE group in both the DG (*F* = 16.20, *p*_SE vs SE + MINO_ < 0.0001) and CA1 (*p*_SE vs SE + MINO_ = 0.0003) subregions by day 7 post‐SE, but then increased dramatically in the CA3 (*F* = 19.09, *p*_SE vs SE + MINO_ = 0.0008) by day 14 and in the CA1 (*F* = 11.43, *p*_SE vs SE + MINO_ = 0.0032) by day 28. Among these spines, minocycline reduced the number of the thin spines in the DG (*F* = 19.44, *p*_SE vs SE + MINO_ < 0.0001) and CA1 (*F* = 22.41, *p*_SE vs SE + MINO_ < 0.0001) subregions in comparison with those in the SE group by day 7 post‐SE. Moreover, after minocycline treatment, the number of thin spines was increased in the CA1 subregion by day 28 post‐SE (*p*_SE vs SE + MINO_ = 0.0265). In the CA3 subregion, significant differences in the numbers of stubby (*p*_SE vs SE + MINO_ = 0.0388), branched (*p*_SE vs SE + MINO_ = 0.0206), and filopodia (*p*_SE vs SE + MINO_ = 0.0036) spines between SE and MINO groups by day 7 were also presented. And the number of mushroom spines was increased in the MINO group by day 14 (*p*_SE vs SE + MINO_ = 0.0149). The significant increase in stubby spines (*F* = 3.664, *p*_SE vs SE + MINO_ = 0.0289) and decrease in branched spines (*p*_SE vs SE + MINO_ = 0.0228) in the CA3 subregion in the MINO group were noted by day 28 post‐SE.

These results indicated that the number of dendritic spines was increased in the DG, CA1, and CA3 subregions in the acute period, but were lost in the CA3 subregions in the transitional period, and then continued to be lost in the DG and CA1 subregions by day 28 post‐SE. Moreover, changes in the number of thin spines were similar to those of the total spines. Importantly, the impairment of dendritic spines by SE could be partly rescued by minocycline.

### 3.4 Ultrastructural Changes in the Synapse

**FIGURE 6:**
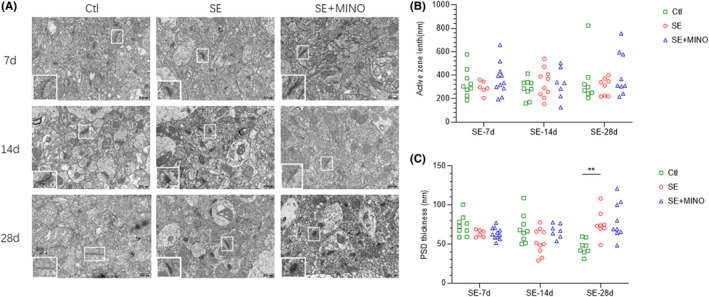
Representative transmission electron microscopy images revealing the ultrastructure of synapses in the hippocampus of the immature mice (30,000×, scale: 500 nm). The areas enclosed by a white box in each picture were amplified to delineate the synapse. (A) The ultrastructure of synapses on the 7th, 14th, and 28th days after KA‐induced SE. (B) The length of the AZ in the presynaptic membrane. (C) The thickness of PSD. One‐way ANOVA and Kruskal–Wallis test followed by Dunnett's post hoc test were used, **p < 0.01. AZ, active zone; PSD, postsynaptic density; SE, status epilepticus.

The functional structure of the presynaptic and postsynaptic membrane were further observed using TEM (Figure 6). The length of the active zone (AZ) of the presynaptic membrane and thickness of the postsynaptic density (PSD) in each group was measured and analyzed using image J software. No difference in the length of the AZ after SE was observed compared with that in the Ctl group. The thickness of the PSD from day 7 to 14 post‐SE tended to decrease in comparison with that in the Ctl group, but these trends were not statistically significant (day7: *F* = 3.451, *p*_Ctl vs SE_ = 0.0860; day14: *F* = 3.044, *p*_Ctl vs SE_ = 0.0562). Furthermore, comparing with that in the Ctl group, a significant increase in the thickness of the PSD by day 28 post‐SE (*F* = 8.929, *p*_Ctl vs SE_ = 0.0056) was presented. However, no difference was observed in the length of the AZ or in the thickness of the PSD between the SE and MINO groups.

### 3.5 Morris water maze

**FIGURE 7:**
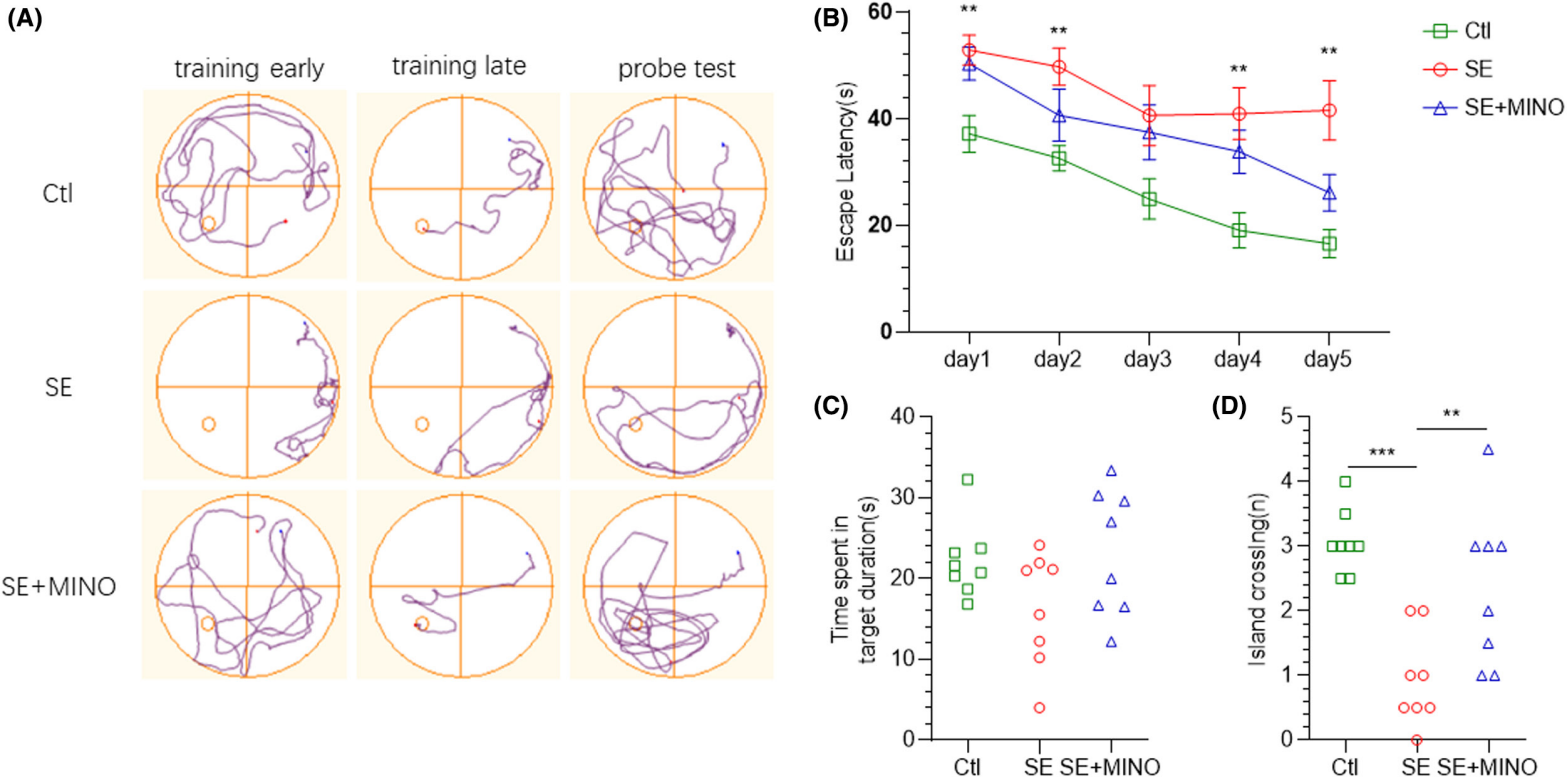
Effects of minocycline on spatial learning and memory in KA‐induced epileptic mice in the MWM test. (A) Representative tracks on training day 1, day 5, and probe test. (B) The average escape latency to the hidden platform location for each spatial learning day. Two‐way ANOVA followed by Dunnett's post hoc was applied. (C) The time spent in the target quadrants was conducted 24 h after the last learning trial. One‐way ANOVA followed by Dunnett's post hoc was used. (D) The number of entries into the island zone. One‐way ANOVA followed by Dunnett's post hoc test was applied. **p < 0.01, ***p < 0.001. KA, kainic acid; MWM, Morris water maze.

To assess the impact of minocycline on the cognitive ability of SE mice, we tested the spatial learning and memory of the mice on the 28th day after SE by using the MWM (Figure 7). The average latencies were much longer in the epileptic mice in comparison with those in the Ctl group on each day during the acquisition trials (*F* = 9.791, *p*_day1_ = 0.0066, *p*_day2_ = 0.0027, *p*_day3_ = 0.0691, *p*_day4_ = 0.0051, *p*_day5_ = 0.0044).

After 5 days of training, the number of crossing the island of the SE group was significantly lower than that of the Ctl group (*F* = 12.49, *p*_Ctl vs SE_ = 0.0002). Compared with the SE group, significant more island crossing was recorded for the MINO group (*F* = 12.49, *p*_SE vs SE + MINO_ = 0.0063). However, there was no significant difference in the time spent in the target quadrant between the Ctl and SE groups or between the MINO and SE groups.

These findings indicated that epileptic mice showed significant learning and memory impairment in comparison with the Ctl mice. Treatment with minocycline did not markedly affect the changes in the behavior of the epileptic mice over 5 days, but significantly improved the long‐term spatial memory ability in the epileptic mice.

## 4 DISCUSSION

### 4.1 Alterations of M1/M2 microglia induced by SE

It is believed that M1 microglia have proinflammatory effects that impede brain repair and regeneration, whereas M2 microglia exert anti‐inflammatory and prohealing abilities that improve brain repair and regeneration.^30^ Although it has been suggested in recent years that the M1/M2 dichotomy, which only represents two extreme reactive status, is an oversimplified conceptual framework, it still retains values in accessing microglia activity in brain diseases and exploring new therapeutic approaches.^30^ The M1/M2 polarization has been identified in neuroinflammation and neurological disorders.^31^ Drugs and molecules targeting M1/M2 microglia modulation alleviated neuronal damage in neurological disorders,^30^ and M1/M2 microglia modulation may be an essential direction for therapy. Therefore in this study, the M1/M2 paradigm was used to profile the only two extreme immune status of immature epileptic brains with the aim of investigating reactive microglia and examining the effects of minocycline on modulating the M1 and M2 microglia. The experimental results indicated that the proportion of M1 and M2 microglia shifted after SE. The high level of M1 microglia suggests that inflammation plays an essential role by day 7 post‐SE. Moreover, the dynamic alteration of M2 microglial levels indicated their neuroprotective effects were quickly initiated after SE, but weakened later. The alteration of M1 and M2 cells was associated with impairments of dendrites by days 7 and 14 post‐SE.

The homeostasis of the CNS is dependent on the exchange of signals between microglia and neurons. This communication is essential for brain functions, including development, immune responses, neuronal circuit remodeling, and plasticity.^32^ It has been reported that the primary function of microglia CXCR3 is microglia recruitment to CXCR3 ligands, leading to dendrite loss and cognitive deficits after brain injuries.^33^, ^34^ In an Alzheimer's disease model, the absence of CXCR3 was associated with attenuated microglial reactivation, reduced inflammatory factors, and improved cognition.^34^ CXCR3 expression on M1 and M2 microglia increased significantly on the 7th day after SE, but declined markedly by day 14 post‐SE, accompanied by alterations in microglial reactivation. These findings revealed that CXCR3 signaling activation and microglial recruitment were both impaired later following SE. Neurons act on microglial CD200R by expressing CD200 and delivering signals to microglia, thereby downregulating microglia inflammatory reactivation and switching to proreparative microglia reactivation.^35^ In this study, microglial CD200R expression on M2 cells increased dramatically after acute SE, indicating a possible rapid protective reaction. However, it tended to decrease significantly later, implying that an inflammatory status still existed. As the receptor of CX3CL1, CX3CR1 is almost exclusively expressed by microglia in the brain. It was reported that reduced dendritic complexity and delayed maturation of newborn neurons were observed in adult *Cx3cr1*‐knockout mice following KA‐induced SE.^36^ A considerable increase in CX3CR1 expression on M2 cells was observed on the 7th day after SE in this study, which was in accordance with the increase in CX3CR1 in animal brains within 7 days after SE reported before.^37^, ^38^ These findings suggested that CX3CL1‐CX3CR1 signaling is activated in response to acute SE. Moreover, previous reports^37^, ^38^ also showed the infusion of anti‐CX3CR1 antibodies to inhibit the signaling could alleviate the neuronal damage caused by SE. In addition, CX3CR1 expression was increased on M1 cells but decreased on M2 cells by day 14 post‐SE, implying that impairment of CX3CR1 in SE persisted. CD68, a lysosomal/endosomal‐associated membrane glycoprotein, is regarded as phagocytic microglial marker. It is reported that CD68 expression on reactive microglia increases in the adult mouse brain after acute KA induced‐SE.^18^ In this work, CD68 expression on M2 cells was dramatically enhanced at the beginning and then decreased markedly by day 14 post‐SE, suggesting that M2 microglia phagocytosis was boosted initially, but inhibited subsequently.

### 4.2 Changes to dendrites and synapses

Dendrite spines serve an essential role in the pathogenesis and development of epilepsy and its morbidities.^39^ Alterations in the number and morphology of dendrite spines occur during both development and adulthood, to establish and remodel neuronal circuits.

In this study, the total numbers of spines in all subregions increased significantly, and the dendritic complexity in the DG increased markedly on the 7th day after SE. Especially, the dendritic complexity in the DG subregion continued to increase significantly to that on the 14th day after SE. Accompanying these changes, there was an increase in the level of SYP that continued by day 14 post‐SE, which has a key function in synaptic vesicle trafficking.^40^ Taken together, these results indicated that spine transitional increases after acute SE, with some selective continuation by day 14 post‐SE. It was reported that the pilocarpine‐induced seizure activity elevated the complexity of dendritic spines, and kainic acid‐induced non‐NMDA receptor activation promotes dendrite growth, in particularly, primary dendrite number and length, from immature cortical neurons in vitro.^41^, ^42^ Additionally, the number of thin spines, which carry small or immature synapses and accommodate changed inputs,^43^ was increased dramatically in all subregions after acute SE in this study. According to previous researches, the initial limbic seizures increase hippocampal neurogenesis, including an increase in newly born neurons.^41^, ^44^, ^45^, ^46^ In addition, epileptiform bursts of newly born granule‐like cells and CA3 pyramidal cells have been observed, suggesting new mechanisms that could contribute to abnormal excitability.^45^ These findings reflect aberrant neurogenesis and dendrites, which might lead to SE‐related abnormal synaptic connections.

It was reported that microglia could eliminate synapses by phagocytic engulfment of dendritic spines and axon terminals.^47^ M2 Microglia reactivation with increased expression of CX3CR1 and CD68 after acute SE indicated that M2 microglia plays an important role in the elimination of spines. However, it seemed to be inconsistent with the increase in dendritic spines and synaptic proteins. It might be explained by the fact that the proportion of M2 microglia with phagocytic activity was smaller in microglia.

By day 14 post‐SE, the total number of spines and the dendritic complexity showed considerable reductions in comparison to the Ctl group in both the CA1 and CA3 subregions. During this stage, CD68 and CD200R expression on M2 microglia decreased dramatically, following the significant decline of the M2 microglial proportion. This implied that the ability of phagocytosis and anti‐inflammation of M2 microglia were both reduced in the time‐point. In the pilocarpine rodent epilepsy model, dendritic spine density in the CA1 and CA3 subfields was selectively decreased after SE, whereas inflammatory parameters were enhanced significantly.^48^ It is proposed that inflammation might lead to dendritic spine loss after SE. Inflammation, such as lipopolysaccharide exposure, viral immune activation, and extended exposure to inflammatory extracellular vesicles, was also shown to cause a significant decrease in dendritic spine density in hippocampal neurons and hippocampal slices in vivo and in vitro.^49^, ^50^, ^51^

On the 28th day after SE, the dendritic complexity was low in all subregions. The total numbers of spines in the DG and CA1 subregions decreased, with the most significant effects observed for the thin spines. The level of SYP became low. These findings revealed the direct effects of seizures on the loss of dendrites and synaptic injuries by day 28 post‐SE. However, it seemed to have no association with M1 and M2 microglia, because no alteration was observed for reactive microglia. Dendrite growth inhibition might be a strategy by which developing neurons compensate for the network hyperexcitability of epilepsy.^52^ However, it remains unknown whether these changes are the cause or the result of epilepsy.

### 4.3 Effects of minocycline

We found that minocycline ameliorated the impaired cognition in a KA‐induced epilepsy model. The MWM results revealed that 3 days of treatment with minocycline during acute SE could mitigate the impairment of spatial learning and memory by day 28 post‐SE. The improvement by minocycline is in agreement with previous studies on KA‐induced epileptic rats,^53^ Fragile X Syndrome,^19^ and sleep deprivation.^21^ It has been demonstrated that minocycline could decrease the number of reactivated microglia in SE models induced by KA and pilocarpine.^16^, ^17^ However, few studies related to epilepsy have focused on the effects of minocycline on M1/M2 microglial reactivation. In this study, minocycline could inhibit M1 microglia reactivation at the three time points and promote M2 microglia reactivation after acute SE, indicating the anti‐inflammatory and neuroprotective effects of minocycline on KA‐induced brain injury by switching the M1/M2 phenotype. Inhibition of microglial reactivation has been reported in many models of CNS diseases.^54^ First, minocycline suppresses the phagocytosis activity of microglia to promote the survival of newborn cells in SE.^18^ Besides, minocycline could also inhibit synaptic pruning by decreasing the expression of CD68. Low CD68 expression after minocycline treatment was always observed in M1 and M2 microglia after SE, indicating that minocycline inhibits microglial phagocytosis. Second, via its combined effects on the enhanced expression of microglial CD200R, the decreased expression of microglial CXCR3 and M1/M2 switching, minocycline might promote neuronal survival by inhibiting M1 mediated proinflammation and promoting M2 neuroprotection.^55^

Minocycline reversed the damage to dendritic spines and SYP protein levels caused by KA‐induced SE in immature mouse brains by days 7, 14, and 28 post‐SE. Accompanying with these alterations, minocycline increased the expression level of PSD‐95 by days 14 and 28 post‐SE. Minocycline treatment could not only enhance the dendritic length and branching,^19^ but also induces the maturation of dendritic spines in brain injuries.^56^ In this study, minocycline increased the dendritic number and complexity in the CA3 subregion after SE, and selectively increased those parameters in DG and CA1 subregions on the 14th and 28th day after SE. Interestingly, minocycline reduced dendritic numbers and complexity in the DG subregion by days 7 and 14 post‐SE, as well as decreasing the number of dendrites in the CA1 subregion after acute SE. As we know, Matrix metalloproteinase 9 (MMP‐9), which was found to increase in epileptic models and patients, contributed to inflammatory reactions and synaptic plasticity.^57^ It is reported that MMP‐9 deficiency not only diminished pruning of dendritic spine, but also reduced aberrant synaptogenesis following mossy fiber sprouting.^58^ Similarly, in the models of Fragile X Syndrome, the synaptic over‐growth and overelaboration displayed in the dFMR1 KO were prevented by minocycline treatment.^59^ Therefore, minocycline, an inhibitor of MMPs,^60^ might through inhibiting MMP‐9 exert its complicated effects on dendrites after SE. In this study, minocycline selectively enhanced or inhibited the number and complexity of dendritic spines in the different subregions of the hippocampus during different stages following SE, implying a complicated mechanism of minocycline in immature brain injury caused by KA, which requires further exploration.

## 5 CONCLUSION

In this study, we reported the immune status of the immature epileptic brain and microscopic modifications in brain structure at the dendritic level throughout the period of epileptogenesis in a KA‐induced SE model. Minocycline treatment could ameliorate the cognitive deficits in immature epileptic mice, accompanied by alterations at the microglial and dendritic levels. Taken together, our findings provided potential mechanistic insights into behavioral and cognitive changes caused by KA‐induced SE, as well as the impact of minocycline on these changes.

## AUTHOR CONTRIBUTIONS

LLX and LJ designed the study. LLX, XYY, TYL, JL, HS, LC, XJS, and WH performed the experiments and collected the data. ZYH and HYL performed animal handling. XYY drafted and finalized the manuscript. XYY and LLX edited the manuscript. LLX and LJ performed the supervision. All authors read and approved the final manuscript.

## CONFLICT OF INTEREST STATEMENT

All authors declare no conflict of interest.

## Supporting information


Figure S1.

Figure S2.

Figure S3.

Figure S4.

Table S1.

## Data Availability

The data that support the findings of this study are available from the corresponding author upon reasonable request.
