# Supplementary material for: Effects of minocycline on dendrites, dendritic spines, and microglia in immature mouse brains after kainic acid‐induced status epilepticus
Source: CNS Neurosci Ther. 2023 Jul 12;30(2):e14352. doi: 10.1111/cns.14352 (PMC10848062; doi:10.1111/cns.14352)
Supplement: Supplementary file 1 — Figure S1. [file CNS-30-e14352-s004.docx]

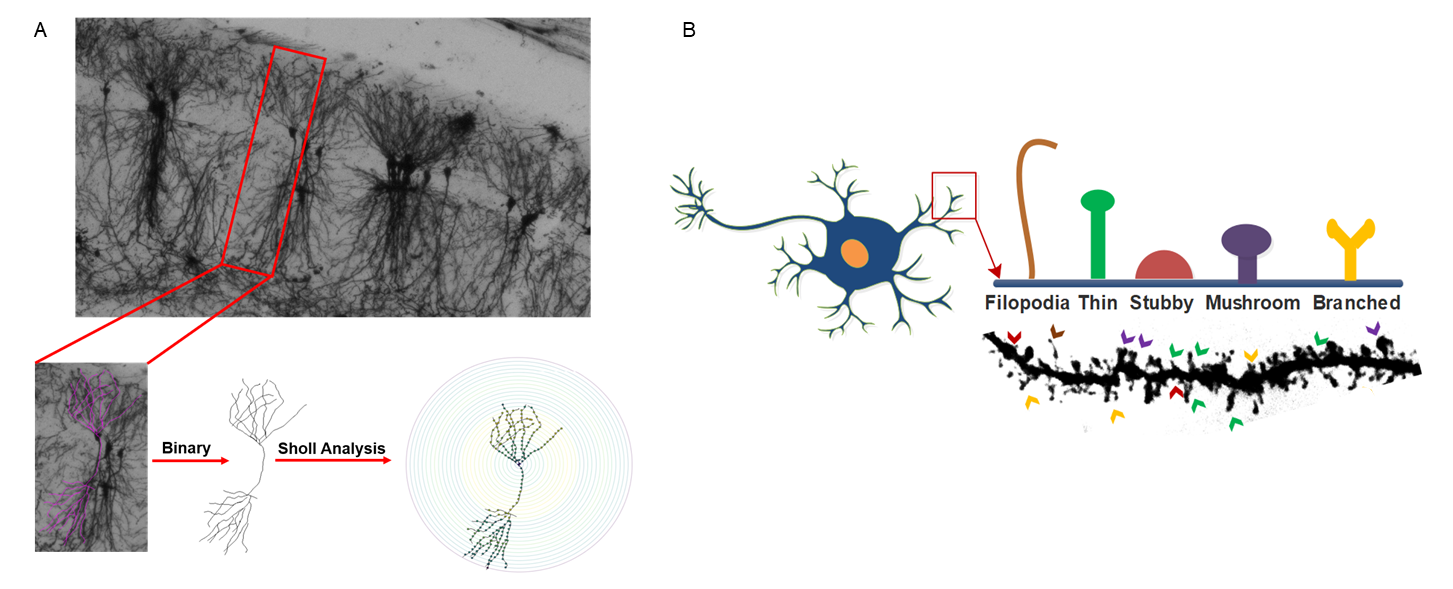


# Figure S1. Sholl analysis and calculation of dendritic spines. (A) Sholl Analysis for the intersection of dendrites and the concentric circles; (B) Calculation of the morphology of dendritic spines: green for thin spines, purple for mushroom spines, red for stubby spines, yellow for branched spines, and brown for filopodia spines.
