# Supplementary material for: Effects of minocycline on dendrites, dendritic spines, and microglia in immature mouse brains after kainic acid‐induced status epilepticus
Source: CNS Neurosci Ther. 2023 Jul 12;30(2):e14352. doi: 10.1111/cns.14352 (PMC10848062; doi:10.1111/cns.14352)
Supplement: Supplementary file 2 — Figure S2. [file CNS-30-e14352-s003.docx]

#
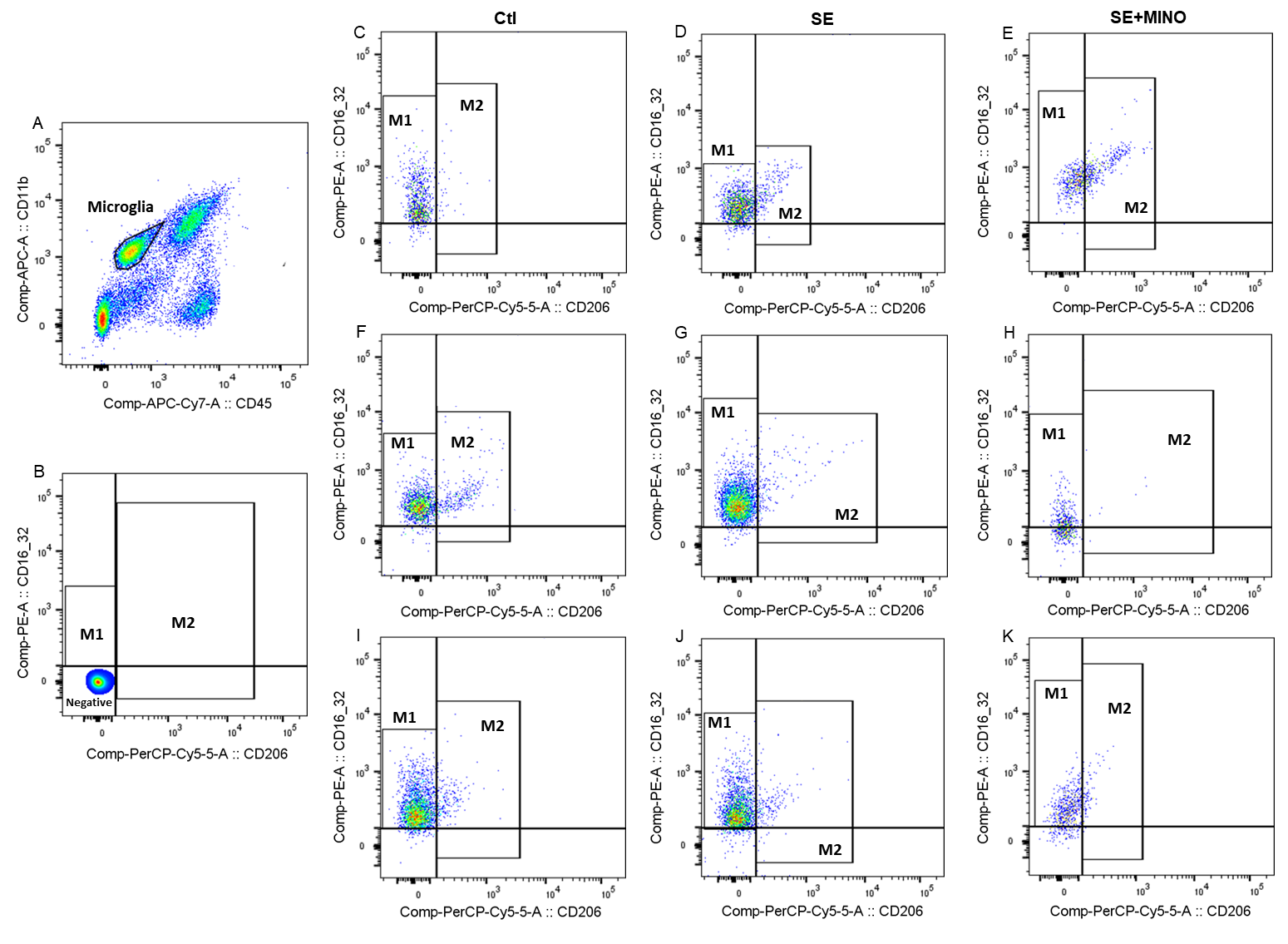


# Figure S2. Alteration of microglia from brain tissues at different time points after KA-induced SE and SE with MINO treatment. (A) Microglia were stained using the flow cytometry markers CD45-APC/Cy7^+^CD11b-APC^+^ to quantify the total CD11b^+^/CD45^int^ microglia; (B)The gating strategy was defined by negative isotype controls, CD16/32-PE^+^CD206-PerCP/Cy5^-^ M1 and CD206-PerCP/Cy5^+^ M2; (C, F, I) Mice receiving 0.9% saline and diazepam were used as controls for the SE group (on the 7th, 14th, and 28th day after SE). (D, G, J) The percentages of M1 and M2 microglia were calculated on the 7th, 14th, and 28th day after SE; (E, H, K) The percentages of M1 and M2 were calculated on the 7th, 14th, and 28th day after SE with MINO treatment. SE: status epilepticus; MINO: minocycline. KA, kainic acid; SE, status epilepticus; MINO, minocycline; CD45, CD45 molecule; APC, Allophycocyanin; Cy7, Cyanine 7; CD11b, integrin subunit alpha M; CD16/32, Fc gamma receptor IIIa; PE, Phycoerythrin; CD206, mannose receptor C-type 1; PerCP, Peridinin chlorophyll protein complex; Cy5, Cyanine 5.
