# Supplementary material for: Effects of minocycline on dendrites, dendritic spines, and microglia in immature mouse brains after kainic acid‐induced status epilepticus
Source: CNS Neurosci Ther. 2023 Jul 12;30(2):e14352. doi: 10.1111/cns.14352 (PMC10848062; doi:10.1111/cns.14352)
Supplement: Supplementary file 3 — Figure S3. [file CNS-30-e14352-s001.docx]

#
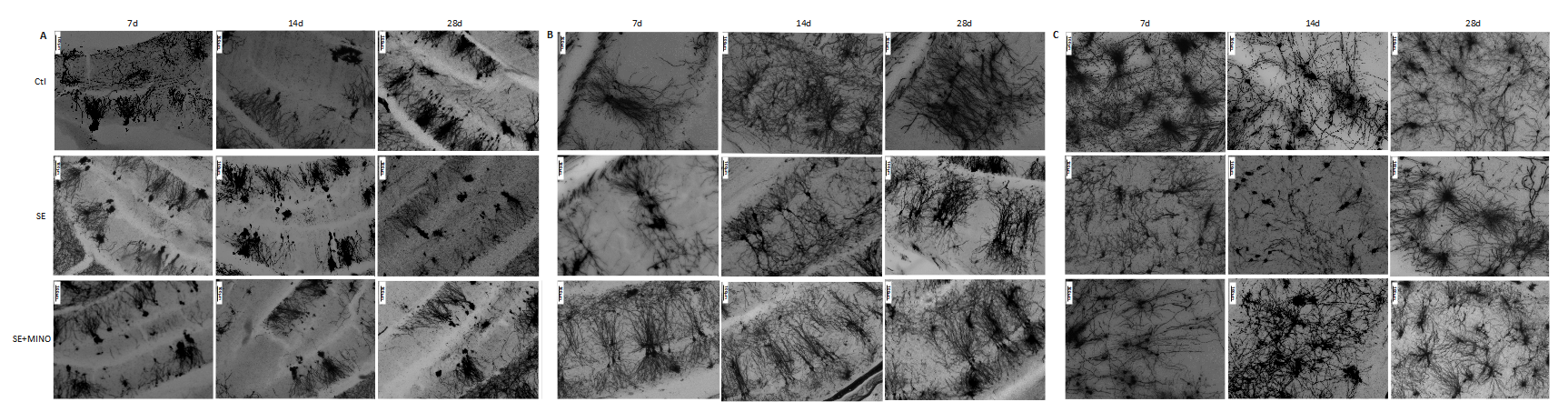
Figure S3. Morphology of dendrites in the DG, CA1, and CA3 subregions of the hippocampus by the 7^th^, 14^th^, and 28^th^ day after KA-induced SE. (A) Morphology of dendrites in DG subregions. (B) Morphology of dendrites in CA1 subregions. (C) Morphology of dendrites in CA3 subregions. KA, kainic acid; SE, status epilepticus; DG, dentate gyrus CA1, cornu ammonis 1; CA3, cornu ammonis 3.
