# Supplementary material for: Effects of minocycline on dendrites, dendritic spines, and microglia in immature mouse brains after kainic acid‐induced status epilepticus
Source: CNS Neurosci Ther. 2023 Jul 12;30(2):e14352. doi: 10.1111/cns.14352 (PMC10848062; doi:10.1111/cns.14352)
Supplement: Supplementary file 4 — Figure S4. [file CNS-30-e14352-s002.pdf]

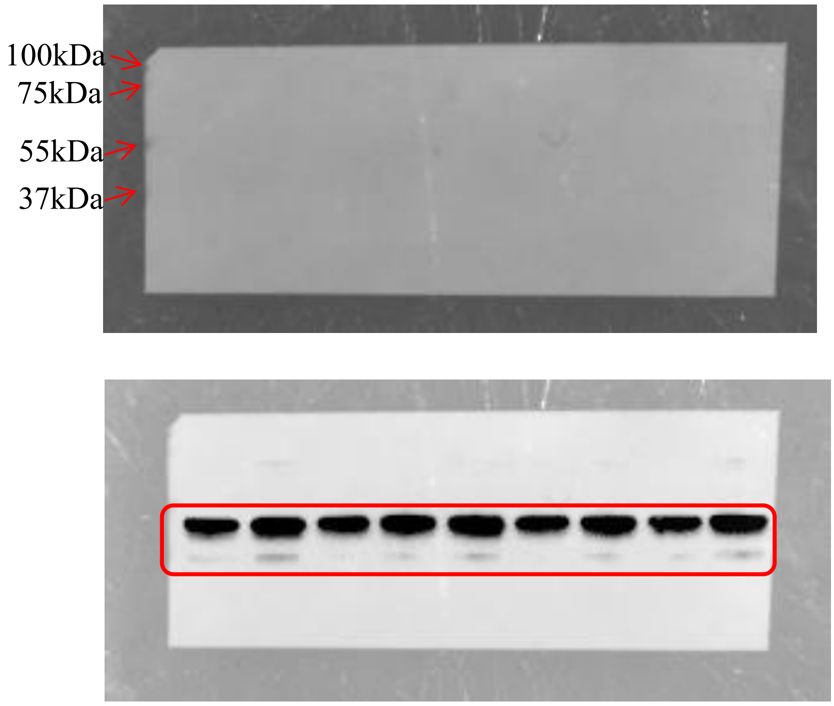

SYP-Full unedited gel/blot for Figure 1

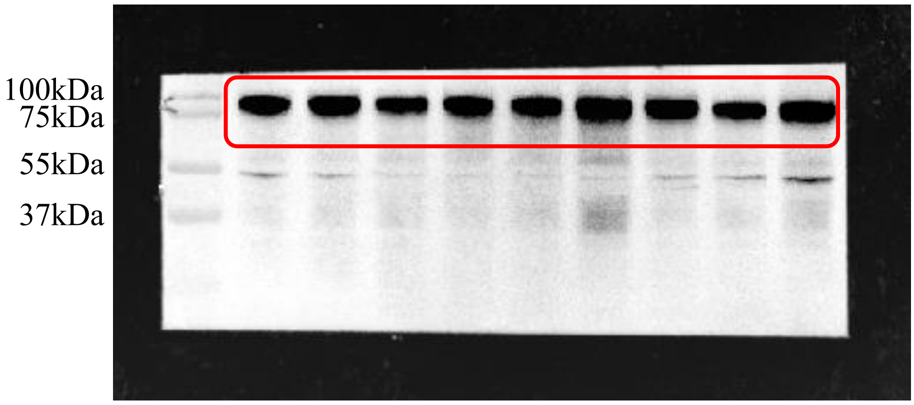

PSD95-Full unedited gel/blot for Figure 1

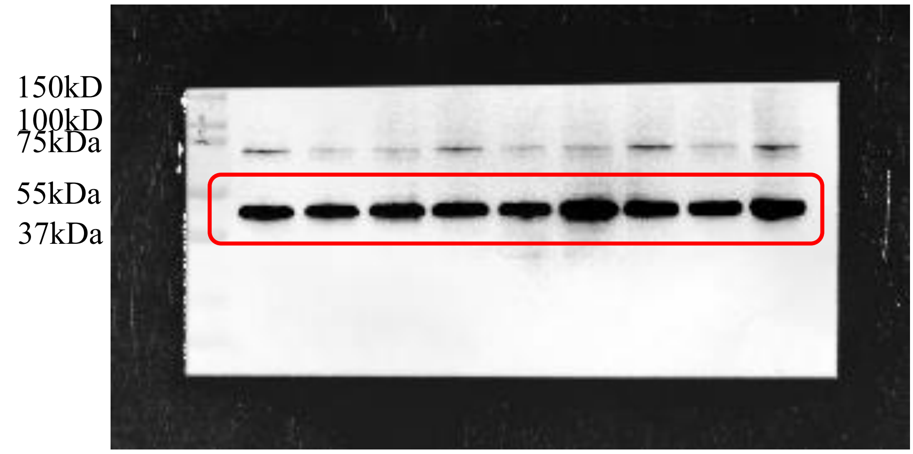

GAP43-Full unedited gel/blot for Figure 1

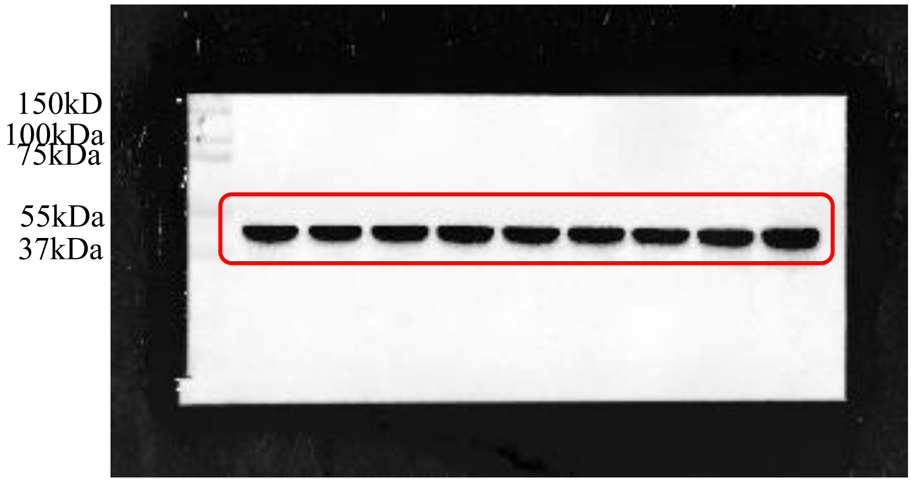

β-actin-Full unedited gel/blot for Figure 1
