# Supplementary material for: Effects of minocycline on dendrites, dendritic spines, and microglia in immature mouse brains after kainic acid‐induced status epilepticus
Source: CNS Neurosci Ther. 2023 Jul 12;30(2):e14352. doi: 10.1111/cns.14352 (PMC10848062; doi:10.1111/cns.14352)
Supplement: Supplementary file 5 — Table S1. [file CNS-30-e14352-s005.docx]

| Antibody | Isotype | Antibody Type | Reactivity | Concentration  (mg/ml) | Volume  (µl per 10^6^ cells in 100 µl volume) |
| --- | --- | --- | --- | --- | --- |
| CD45-APC/Cy7 | Rat IgG2b, κ | Monoclonal | Mouse | 0.2 | 1.0 |
| CD11b-APC | Rat IgG2b, κ | Monoclonal | Mouse | 0.2 | 1.0 |
| CD16/32-PE | Rat IgG2a, λ | Monoclonal | Mouse | 0.2 | 1.0 |
| CD206-PerCP/Cy5.5 | Rat IgG2b, κ | Monoclonal | Mouse | 0.2 | 1.0 |
| CX3CR1-PE/Cy7 | Mouse IgG2a, κ | Monoclonal | Mouse | 0.2 | 0.1 |
| CXCR3-Brilliant Violet 510 | Armenian Hamster IgG | Monoclonal | Mouse | 0.2 | 1.0 |
| CD68-Brilliant Violet 605 | Rat IgG2a | Monoclonal | Mouse | 0.2 | 1.0 |
| CD200R-FITC | Rat IgG2a, κ | Monoclonal | Mouse | 0.5 | 1.0 |

Table S. Detailed information of antibodies for flow cytometric staining
